# Supplementary material for: Functional Study of cAMP-Dependent Protein Kinase A in Penicillium oxalicum
Source: J Fungi (Basel). 2023 Dec 16;9(12):1203. doi: 10.3390/jof9121203 (PMC10745023; doi:10.3390/jof9121203)
Supplement: Supplementary file 1 [file jof-09-01203-s001.zip › jof-2734363-supplementary.pdf]

Supplementary Material

Journal Name: *Journal of Fungi*

Article Title: Functional study of cAMP-dependent protein kinase A in *Penicillium oxalicum*

Authors: Qiuyan Sun[1], Gen Xu[1], Xiaobei Li[1], Shuai Li[1], Zhilei Jia[1], Mengdi Yan[1], Wenchao Chen[1], Zhimin Shi[1], Zhonghai Li1*, Mei Chen1**

*** Correspondence:**
Zhonghai Li, lzhlzh@vip.126.com, Qilu University of Technology, Shandong Academy of Sciences, Jinan, 250353, P. R. China

Mei Chen, chenmei_16@163.com, Qilu University of Technology, Shandong Academy of Sciences, Jinan, 250353, P. R. China

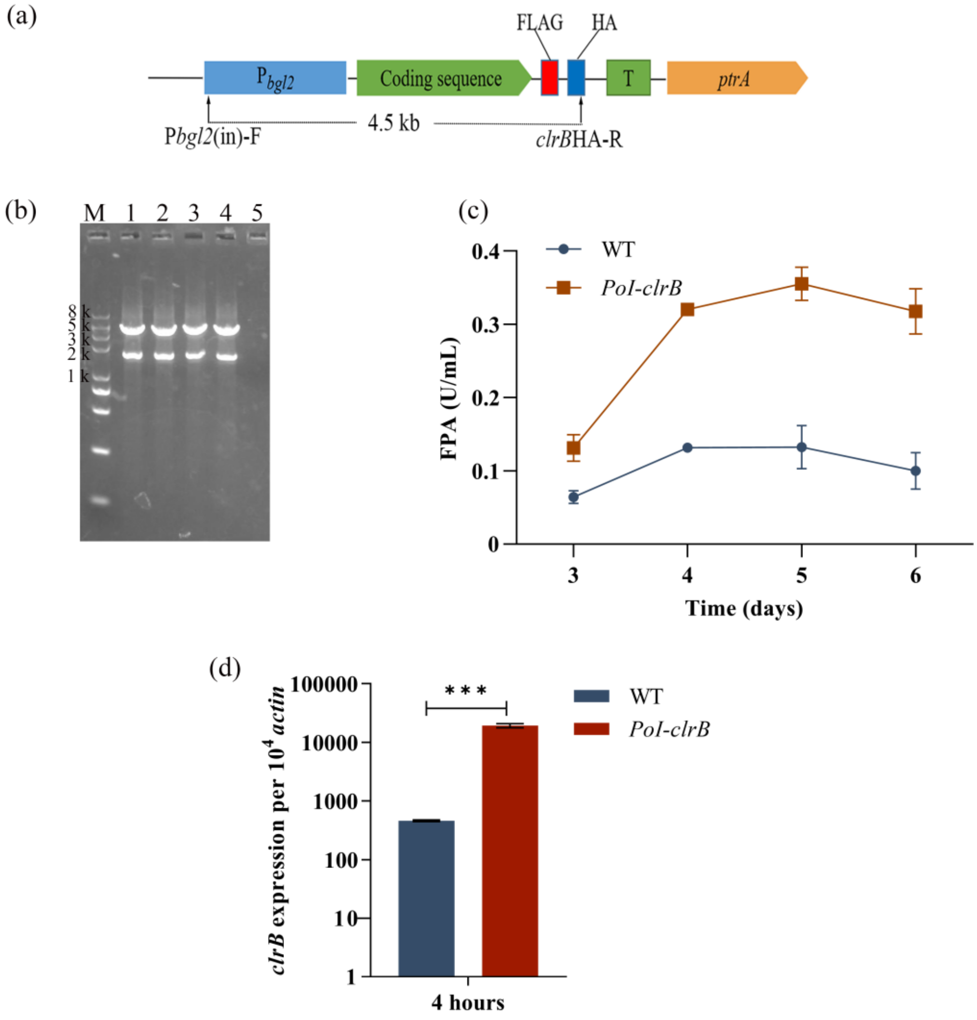

Figure S1. Construction and validation of tagged ClrB overexpression strain (a) Construction strategy of *PoI-clrB* strain. (b) *PoI-clrB* was verified by PCR. *PoI-clrB* was verified by the primer Pbgl (in)-F /clrBHA-R, which was 4.5 kb in size (lanes 1-4); Lane 5 is the *P. oxalicum* 3-15 genome; Lane M is the Trans2K Plus II DNA marker (TransGen Biotech,Beijing, China). (c) Activities of filter paper enzyme of *P. oxalicum* 114-2 and *PoI-clrB*. Activities of filter paper enzyme were measured every 24 hours from 72 hours to 144 hours. (d) RT-qPCR analysis of *clrB* in *P. oxalicum* 114-2 and *PoI-clrB*.

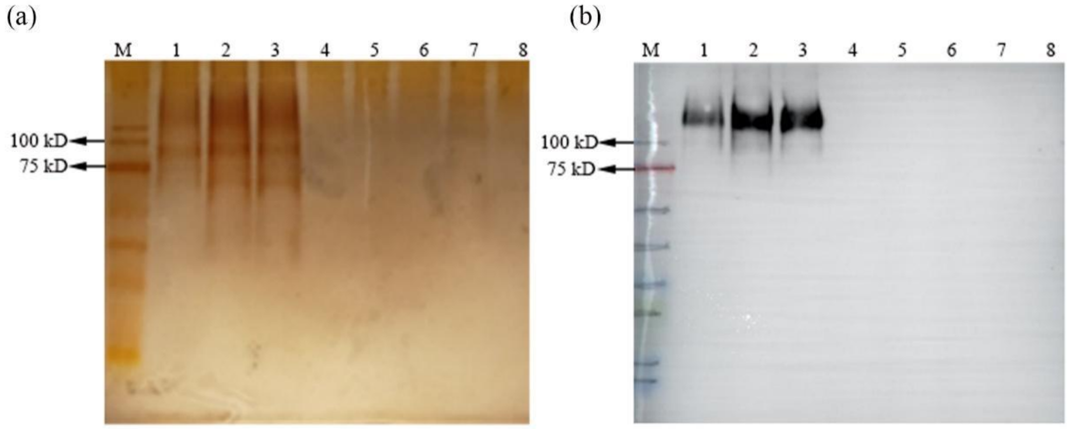

Figure S2. ClrB protein complex silver stain and western blot analysis (a) Results of silver staining. Lanes 1, 2 and 3 are ClrB protein complex anti-Flag eluate; Lanes 4, 5 and 6 are ClrB protein complex anti-HA eluate; Lanes 7 and 8 are *P. oxalicum* 114-2 eluate. (b) Western blot analysis. Lanes 1, 2 and 3 are ClrB protein complex anti-Flag eluate; Lanes 4, 5 and 6 are ClrB protein complex anti-HA eluate; Lanes 7 and 8 are *P. oxalicum* 114-2 eluate.

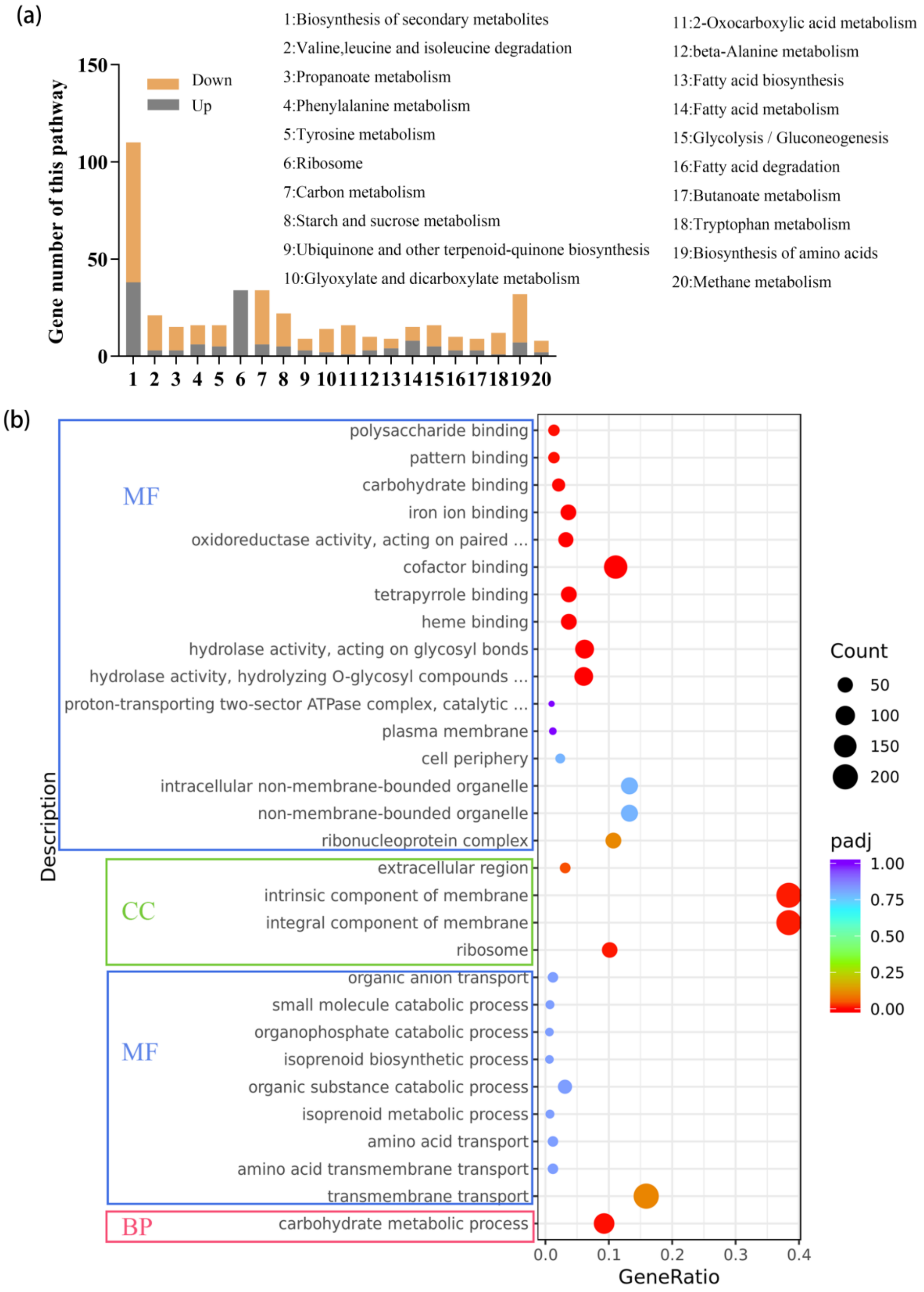

Figure S3. Transcriptome analysis of *P. oxalicum* 114-2 and Δ*PoPKA-C*. (a) KEGG annotation and enrichment analysis were performed for differentially expressed genes. (b) GO enrichment analysis was performed for differentially expressed genes ($P < 0.05$).

Table S1 Names and sequences of primers used in this study

Primer names	Primer sequences
3213-F0	CAGCCTGAGGAATCGGGACG
3213-F1	ATTGCTGGACGGTGGTTAGT
3213-F2	AGCCGTACATCTAATCCACA
3213HPH-R	gctccttcaatatcagttaacgtcg AGGCCATAGACTTTGGAGAA
3213-F	TGAAGAAGCGGCGAACGAAG
3213-R	TTAGTGGGTGGACGAGTGAGGG
3213HPH-F	aaattccgtcaccagccctgggttgGCCCTGTATAATTTATGACG
3213-R2	GAGCCAGACAAGTTTGAACG
3213-R1	ACATCAGCACTGCCAGTATG
hph-F	CGACGTTAACTGATATTGAAGGAGC
hph-R	CAACCCAGGGCTGGTGACGGAATTT
(ptrA)3213-R	ATGGGATCCCGTAATCAATTGCCCCGAATGGCATT GTTCCCGTC
ptrA-F	GGGCAATTGATTACGGGATC
ptrA-R	ATGGGGTGACGATGAGCCGC
PtrA(In)-R	CCGCTCTTGCATCTTTGTTTG
(PtrA)HAClrB-R	GATCCCGTAATCAATTGCCC CCGTTTCCCACATAGCATC
Pbgl2-F	CGCTTGTGCTCGCAGGGATG
Pbgl2-R	CTTGGCGAAGTCGATTGGAAC
Pbgl2(In)-F	TGGCACGGGCTTGATTGAG
(Pbgl2)ClrB-F	CGTTCCAATCGACTTCGCCAAGATGTTCCACACCTT TGAAGG
3213tz-F	GACGGCACGTTCGGCTTGA
RT-act-F	GTTCCATTCTCGCCTCCCTCT
RT-act-R	AGAAGCACTTGCGGTGAACGA
RT-cbh1-F	CCACCACCACTACCAGCAAGG
RT-cbh1-R	GTAGCCAACACCACCGCACT
RT-eg1-F	ACCGCTGCTCAGACCACGAC

RT-eg1-R	TGGGTCCCGAGTAGCCAACG
RT-bgl1-F	CACCAACACCGGCTCAGTTA
RT-bgl1-R	GGACATCCCAGTTGGACAGAT
RT-bgl2-F	GGCTGATGCGTACACGTTTGA
RT-bgl2-R	CGACATAAGTCACGCCGAAGC
RT-creA-F	ACAGTCCTGGTCAAGGTCAC
RT-creA-R	GCCCGCCACGGAATTATTTG
RT-clrB-F	TTGCCCGCATTTACGAAGCC
RT-clrB-R	GTCTTGGGGTCCATTTTCGC
RT-xlnR-F	GTGGTCCGAGCCTGCGAAAC
RT-xlnR-R	CAGCGGTAGAGGGCGAGAAC
RT-brlA-F	GGAACATCTCAAGCGGCACA
RT-brlA-R	CAACTTGGAGCCGTAGATGG
RT-fluC-F	GGTCTGCGACAAGCGGTTCA
RT-stuA-F	GCGGCTCCTACACTTACACC
RT-stuA-R	GAACGGCATCGTCGTCATCG
RT-3213-F	CTCCCATCCTCAGCCCTCTC
RT-3213-R	GGAGTCTGCGATTGCGATTG
